# Free-floating thrombus of the aorta: 3 case reports

**DOI:** 10.1186/s40792-021-01230-7

**Published:** 2021-06-10

**Authors:** Naohiko Oki, Yoshito Inoue, Sohsyu Kotani

**Affiliations:** Department of Cardiovascular Surgery, Hiratsuka City Hospital, 1-19-1 Minamihara, Hiratsuka City, Kanagawa 254-0065 Japan

**Keywords:** Thrombolysis, Aorta, Computed tomography angiography, Embolic events, Free-floating thrombi, Idiopathy, Surgical thrombectomy

## Abstract

**Background:**

Idiopathic free-floating thrombus (FFT) of the aorta is a rare occurrence, but it can lead to catastrophic consequences. The initial symptoms are typically cerebral or peripheral embolisms. Surgical thrombectomy and thrombolysis are two primary treatments for FFT. Here, we report three cases of patients with idiopathic FFT in the absence of coagulopathy who were treated successfully by surgery with no recurrent thrombi or relapse of symptoms.

**Case presentation:**

Case 1 involved a 72-year-old male patient with a pedunculated thrombus in the distal aortic arch. Case 2 involved a 62-year-old female patient with a cylinder thrombus in the aortic arch and left common carotid artery. Case 3 involved a 65-year-old male patient with three pedunculated thrombi in the ascending aorta, aortic arch, and left subclavian artery. None of the patients had clinical signs of coagulopathy. Pedunculated or cylinder thrombi have a greater risk of breaking off, which can produce severe peripheral embolism in contrast with intramural thrombi (73% vs. 12%). Due to the high embolism risk for each patient, conservative medical treatment by heparinization was deemed inappropriate, so each patient underwent emergency surgical thrombus removal. After surgery, each of the three patients was treated with warfarin for secondary prevention of thromboembolism. At 7-month follow-up in outpatient practice, a computed tomography (CT) scan indicated that Patient 1 had no recurrent thrombus, and the patient has been symptom-free for 11 months. At 1-month follow-up in outpatient practice, a CT scan indicated that Patient 2 had no recurrent thrombus, and the patient has been symptom-free for 8 years. At 3-week follow-up in hospital, a CT scan indicated that Patient 3 had no recurrent thrombus, but he failed to follow-up after discharge, so his follow-up status is unknown.

**Conclusions:**

For a large pedunculated or cylinder thrombus located in the thoracic aorta, surgical thrombectomy should be performed. And, in surgical thrombectomy, the location of the cannulas and cross-clamp should be selected carefully according to the location of the thrombus. After surgery, anticoagulant is important to prevent recurrent idiopathic thrombi.

## Background

Free-floating thrombus (FFT) of the aorta develop in the absence of pre-existing disease, is rare, especially in the absence of coagulopathy. The common causes of FFT are coagulopathy, steroid use, trauma, drug abuse, heparin-induced thrombotic thrombocytopenia, rheumatism, primary endothelial disorders, iatrogenesis, and vasculitis [1]. Idiopathic FFT is easily missed due to their low incidence. However, they may cause cerebral and peripheral embolisms, which are often primary symptoms. Occasionally, FFT can be detected by computed tomography angiography (CTA), which is now widely used. The etiopathogenesis remains enigmatic, and the primary management consists of surgery and thrombolysis. Due to the rarity of the condition and need for guidance on successful techniques, we are reporting the surgical experiences of three cases of idiopathic FFT of the aorta.

## Case presentation

**Fig. 1:**
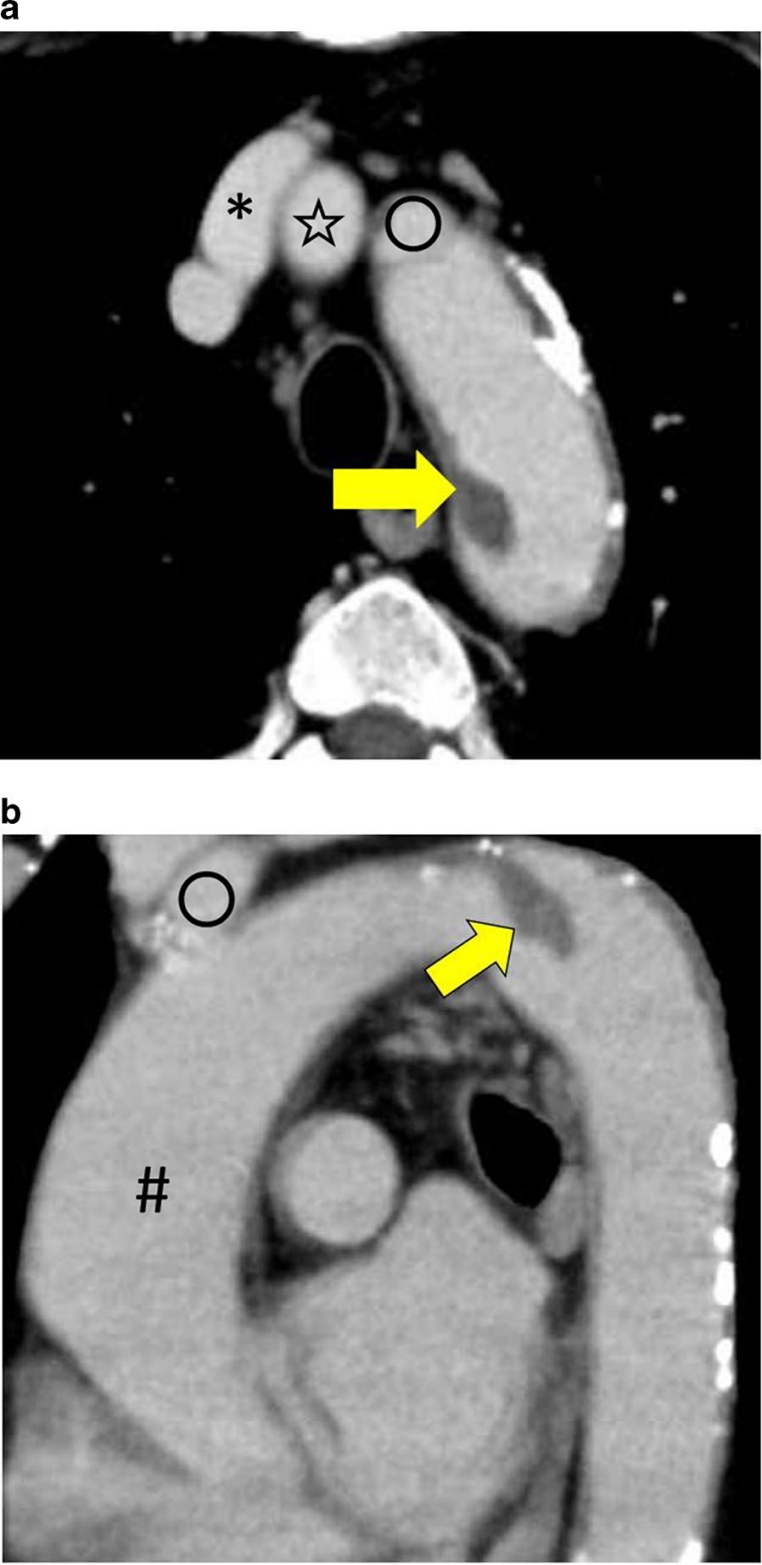
CT images. **a** An axial CT image showing the thrombus in the distal aortic arch (arrow). **b** A sagittal CT image showing the thrombus in the distal aortic arch (arrow). *Brachiocephalic artery, open star left common carotid artery, open circle left subclavian artery, # ascending aorta

**Fig. 2:**
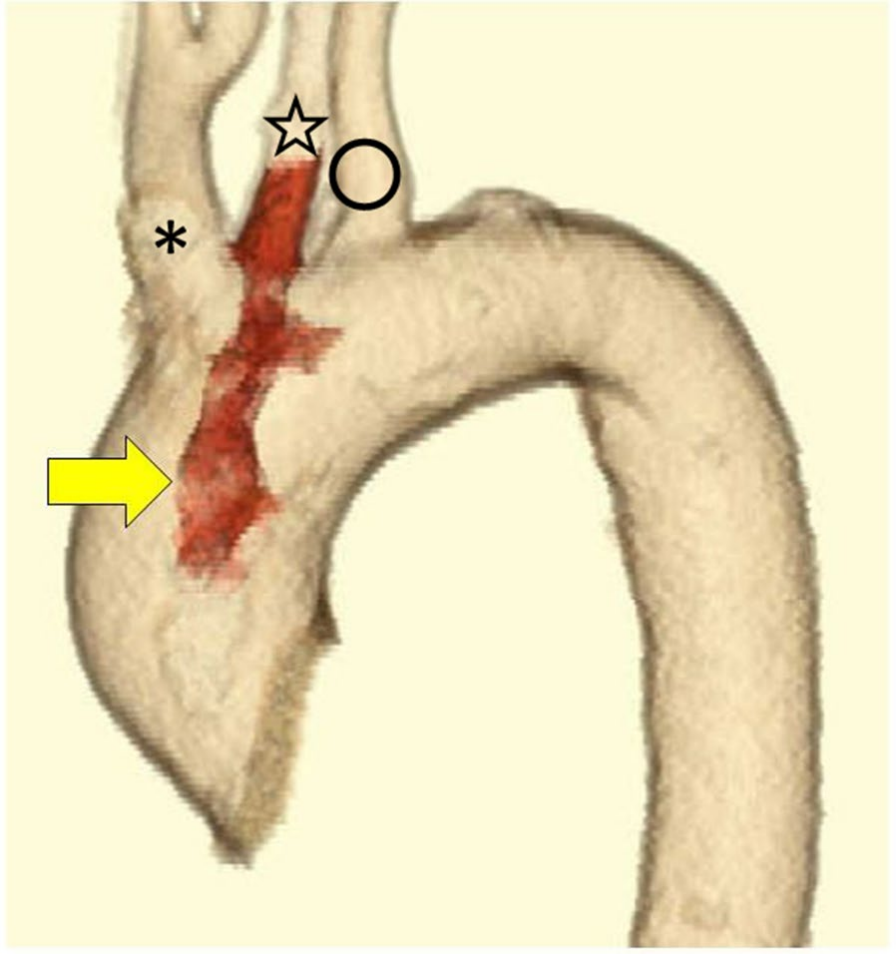
A multiplaner reconstruction CT image showing the cylinder thrombus in the aortic arch and left common carotid artery (arrow). * Brachiocephalic artery, open star left common carotid artery, open circle left subclavian artery

**Fig. 3:**
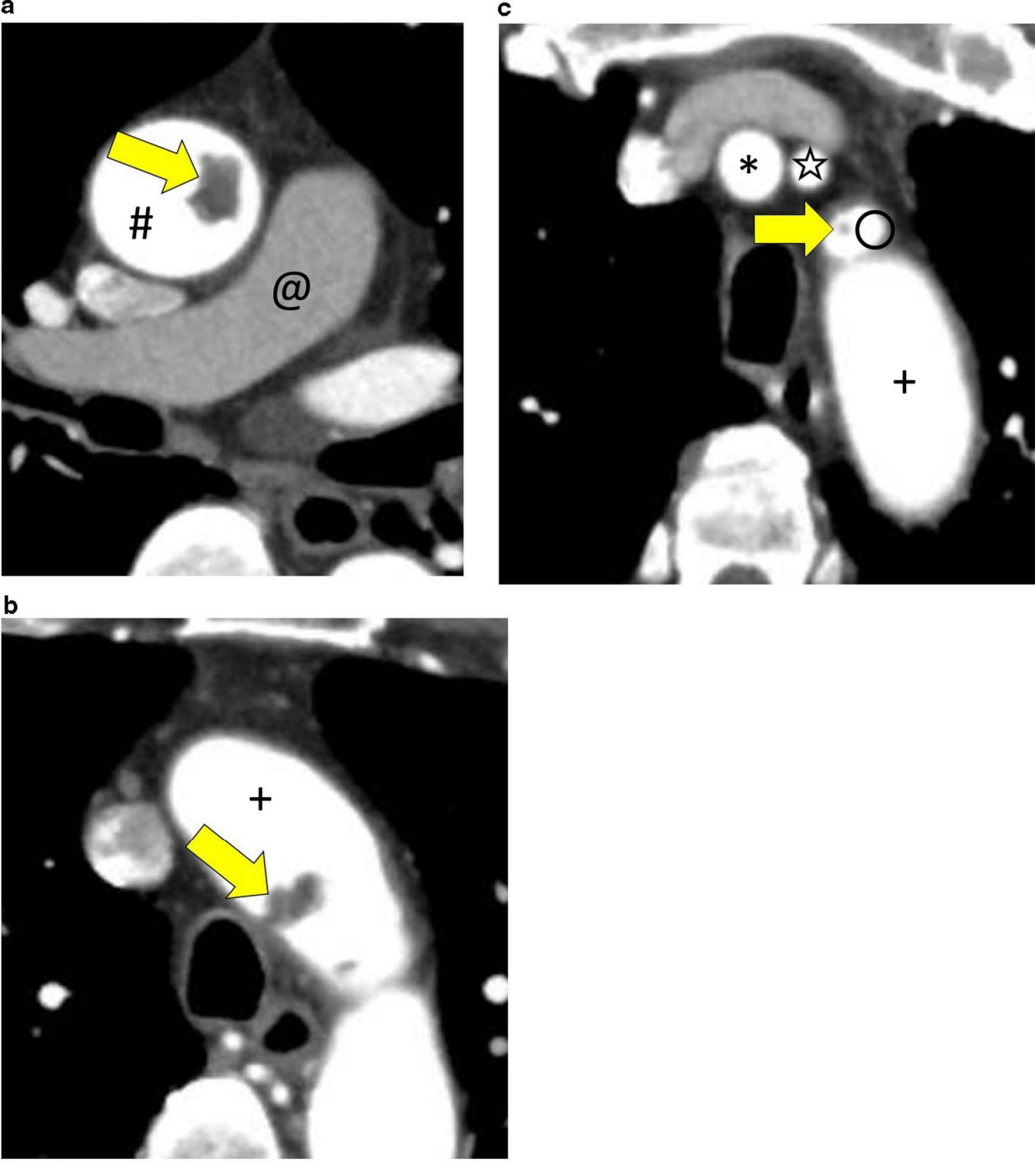
CT images. **a** An axial CT image showing the thrombus in the ascending aorta (arrow). **b** An axial CT image showing the thrombus in the aortic arch (arrow). **c** An axial CT image showing the thrombus in the left subclavian artery (arrow). # Ascending aorta, @ pulmonary artery, + aortic arch, * brachiocephalic artery, open star left common carotid artery, open circle left subclavian artery

The clinical features and initial and follow-up computed tomography angiography (CTA) findings of patients with free-floating thrombus (FFT) from January 2013 to October 2020 in our institution were reviewed retrospectively. The collated date consisted of three patients, including two men and one woman. Table 1 details the clinical features of the three patients. None of the patients had coagulopathy. Anticardiolipin antibodies, protein C, protein S, antithrombin III, fibrinogen and platelet count were normal for all three patients. All patients had a history of smoking. They were all in sinus rhythm. Based on echocardiography examination, none of the patients had intracardiac thrombus. Conservative medical treatment by heparinization immediately appeared inappropriate because morphology of the thrombi is pedunculated or cylinder with high embolism risk, so the patients underwent emergency surgical thrombectomy. The procedures are summarized in Table 2. The patient in case 1 (Patient 1) had a pedunculated thrombus in distal aortic arch (Table 3, Fig. 1a, b). In case 1, left posterolateral thoracotomy was performed, and partial cardiopulmonary bypass was established with the femoral vein inflow from the femoral artery outflow. The aorta was cross-clamped between the descending aorta distally and the aortic arch proximally, and the left subclavian artery was also clamped. Then, a trap-door incision in the distal aortic arch was made, and a fresh friable thrombus was removed from under the left subclavian artery. The surgical techniques used for cases 2 and 3 begin similarly, but then become specialized for the process of thrombus removal. The patient in case 2 (Patient 2) had a cylinder thrombus in the aortic arch and left common carotid artery (Table 3, Fig. 2). The patient in case 3 (Patient 3) had three pedunculated thrombi in the ascending aorta, aortic arch, and left subclavian artery (Table 3, Fig. 3a–c). In case 2 and case 3, median sternotomies were performed and cardiopulmonary bypasses were established with the right atrium inflow from the right femoral artery and the right subclavian artery outflow. In case 2, deep hypothermic circulatory arrest (DHCA) was induced, a trap-door incision was made from the ascending aorta to the left common carotid artery, and a fresh friable thrombus was removed. In case 3, DHCA was induced, and a trap-door incision was made from the ascending aorta to the distal aortic arch, and the fresh friable thrombi were removed.

**Table 1:** Clinical features of the three patients with free-floating thrombi

Case	Age	Sex	Main complaint	Embolic event	Past medical history	Family history of vascular disease
1	72	Male	Melena	No	Hyperuricemia	No
2	66	Female	The color of the left great toe changed to black	Left great toe	Bladder cancer, hypertension	No
3	65	Male	Nausea, vomiting, dizziness	Cerebral	Hypertension, hyperuricemia	No

**Table 2:** Surgical details of three patients with free-floating thrombi

Case	Approach	Arterial cannula	Venous cannula	Sites of clamp	Deep hypothermic circulatory arrest
1	Left posterolateral thoracotomy	Left femoral artery	Left femoral vein	Descending aorta, aortic arch between left common carotid artery and left subclavian artery, left subclavian artery	No
2	Median sternotomy	Right femoral artery, right subclavian artery	Right atrium	Ascending aorta	Yes
3	Median sternotomy	Right femoral artery, right subclavian artery	Right atrium	Ascending aorta	Yes

**Table 3:** Details of the three patients’ free-floating thrombi

Case	Location	Size (mm)	Morphology
1. (Fig. 1)	Distal aortic arch	16 × 27	Pedunculated
2. (Fig. 2)	Aortic arch and left common carotid artery	66 × 18	Cylinder
3. (Fig. 3)	Ascending aorta, aortic arch, left subclavian artery	Max 24 × 13 (ascending aorta)	Pedunculated

After surgery, all three patients were treated with warfarin for secondary prevention of thromboembolism. Patient 1 had no recurrent thrombus for 7 months, as evidenced by CT images, and has been symptom-free for 11 months. Patient 2 had no recurrent thrombus according to a 1-month follow-up CT image and has been symptom-free for 8 years. Patient 3 had no recurrent thrombus based on a 3-week CT image, but he failed to follow-up after discharge, so his follow-up status is unknown.

## Conclusions

In 10,671 autopsies, Machleder et al. reported an incidence of thoracic aortic thrombus at 0.45%, of which 17% had autopsy evidence of distal embolization [2]. Management strategies have largely been dependent on anatomic location as well as on morphologic features of the thrombus [2]. Management of FFT is usually surgical thrombectomy or thrombolysis. It has been reported that pedunculated or cylinder thrombi have a greater risk of breaking off, which can produce severe peripheral embolism in contrast with intramural thrombi (73% vs 12%) [3]. Therefore, for pedunculated thrombi, which carry a high risk of embolism, surgical thrombectomy should be performed. Choukroun et al. identified thrombi located in the ascending aorta and aortic arch as an indication that surgery should be performed to prevent stroke [4]. Furthermore, the location of the outflow cannulas or cross-clamp and whether DHCA needs to be performed should be evaluated carefully to prevent stroke and other peripheral embolisms.

In cases whose risks associated with surgery are comparatively high, thrombolysis has been the primary modality of treatment [5]. However, Fayad reported that aortic thrombus persisted or recurred in 26.4% of the thrombolysis group and in 5.7% of the surgery group (*p* < 0.001), and recurrence of peripheral arterial embolization was seen in 25.7% of the thrombolysis group and 9.1% of the surgery group (*p* = 0.003) [6]. Moreover, thrombi are mostly subacute or chronic by the time they are detected, and subacute and chronic thrombi are not suitable for thrombolysis. Therefore, surgical thrombectomy is an optical treatment. And, after surgery, recurrent idiopathic thrombus must be prevented. Posttherapy treatment also makes significant sense to improve a patient’s prognosis. There is no definitive evidence as to the antithrombotic therapy in the case of aortic thrombosis. We chose warfarin therapy in these three cases. There is no knowing optimal prothrombin time-international normalized ratio (PT-INR). In these three cases, PT-INR is controlled at 2.0, and recurrent thrombi are not seen.

In conclusion, surgical thrombectomy should be performed for large pedunculated or cylinder thrombi located in the thoracic aorta. And, in surgical thrombectomy, the location of the thrombus should determine where to perform the outflow cannulas or cross-clamp and whether DHCA should be induced. After surgery, anticoagulant is important to prevent recurrent idiopathic thrombi.

## Data Availability

Not applicable.

## Abbreviations

FFT: Free-floating thrombus
CT: Computed tomography
CTA: Computed tomography angiography
DHCA: Deep hypothermic circulatory arrest
PT-INR: Prothrombin time-international normalized ratio
